# CX3CL1/Fractalkine: A Potential Biomarker for Liver Fibrosis in Chronic HBV Infection

**DOI:** 10.3390/cimb46090593

**Published:** 2024-09-10

**Authors:** Natalia A. Arsentieva, Zoia R. Korobova, Oleg K. Batsunov, Natalia E. Lyubimova, Valentina V. Basina, Elena V. Esaulenko, Areg A. Totolian

**Affiliations:** 1 Laboratory of Molecular Immunology, Saint Petersburg Pasteur Institute, Mira St. 14, 197101 St. Petersburg, Russia; zoia-korobova@yandex.ru (Z.R.K.); batsunov@gmail.com (O.K.B.); natelu@mail.ru (N.E.L.); eve-gpmu@mail.ru (E.V.E.); totolian@spbraaci.ru (A.A.T.); 2 Department of Immunology, Pavlov First State Medical University of St. Petersburg, L’va Tolstogo St. 6–8, 197022 St. Petersburg, Russia; 3 Department of Infectious Diseases of Adults and Epidemiology, Saint Petersburg State Pediatric Medical University, Litovskaya St., Bldg. 2, 194100 St. Petersburg, Russia; v.basina@mail.ru

**Keywords:** CX3CL1/Fractalkine, chemokines, hepatitis B virus, hepatitis C virus, liver fibrosis

## Abstract

A hepatitis B virus (HBV) infection can progress to chronic hepatitis, leading to liver fibrosis, cirrhosis, and hepatocellular carcinoma. CX3CL1/Fractalkine plays a crucial role in recruiting immune cells that are responsible for protecting against HBV infection. The aim of this study was to measure CX3CL1/Fractalkine concentrations in the blood plasma of individuals infected with HBV and to evaluate the role of this chemokine in the development of liver tissue fibrosis. Our study included patients infected with HBV, patients infected with HCV, autoimmune hepatitis, and healthy donors. We analyzed the CX3CL1/Fractalkine concentrations in blood plasma using the xMAP technology. Our results showed that HBV-infected patients had lower concentrations of CX3CL1/Fractalkine. Furthermore, in HBV-infected patients with severe fibrosis/cirrhosis, we observed significantly lower concentrations of CX3CL1/Fractalkine compared to those with no/mild fibrosis. Our study revealed that CX3CL1/Fractalkine concentrations are significantly associated with the stage of fibrosis in HBV infection. We demonstrated that lowered CX3CL1/Fractalkine concentrations might have prognostic value for predicting fibrosis development in liver tissue. Our findings suggest that decreased concentrations of CX3CL1/Fractalkine are associated with an increased risk of progressive liver fibrosis, indicating the potential of this chemokine as a prognostic biomarker for the development of liver fibrosis.

## 1. Introduction

The hepatitis B virus (HBV) is a non-cytopathic DNA virus with high resistance to environmental factors and high affinity for liver cells. HBV infection can lead to chronic hepatitis; however, the risk usually decreases as the patient’s age increases [1]. A chronic HBV infection leads to liver fibrosis with high risks of cirrhosis and hepatocellular carcinoma [2]. Based on the World Health Organization (WHO) data, there are over 250 million individuals suffering from chronic HBV infection; the annual emergence of this disease comprises around 1.2 million new cases [3]. 

The key role in HBV control belongs to immune factors, i.e., natural killer (NK) cells and T NK cells, monocytes and dendritic cells (DCs), cytotoxic T lymphocytes (CTLs), T helper (Th) 1 cells, and T regulatory cells (Tregs) [4]. Immunity inside the liver can be induced by pattern-recognizing receptors (PRRs), e.g., Toll-like receptors (TLR), RIG-I-, and NOD-like receptors. In addition, hepatocytes can produce interferons when infected by HBV [5]. NK cells, TNKs, and CTLs have potent cytotoxic function to control the development of HBV infection [6]. However, a persistent infection caused by HBV can suppress the effector function of immune system with various mechanisms [7].

To provide their effector functions, immune cells must enter the site of inflammation. Chemokines are responsible for immune cell migration; they are produced by monocytes, endothelial cells, hepatic stellate cells (HSC), and hepatocytes [8]. Hepatocytes are directly targeted by HBV, therefore chemokine production by these cells can be induced when compared to other liver cell types [9]. 

CX3CL1/Fractalkine is the only member of the CX3C chemokine family, it can exist in two forms—free and membrane-bound; its polypeptide chain comprises a protein of 397 amino acids and a transmembrane domain [10]. The latter induces the suppression of tumor cell activity, even in hepatocarcinoma [11]. The main sources of CX3CL1/Fractalkine in the peripheral blood and liver tissue are monocytes in a pro-inflammatory state. CX3CL1/Fractalkine can also be secreted by macrophages, fibroblasts, endothelial cells, and dendritic cells [12]. NK cells, CTLs and Th cells, monocytes, and other types of cells express receptor molecule CX3CR1 [13]. NK cells primarily express CX3CR1 and are activated by CX3CL1/Fractalkine in both their adhesion and migration [14]. 

It has been reported that CX3CL1 expression is closely associated with cancer cell metastasis in prostate, gastric, and breast cancer, as well as in renal and colon carcinoma and hepatocellular carcinoma (HCC) in spinal metastases [15].

CX3CR1 has been recognized as a fusion coreceptor for human immunodeficiency virus-1 [16], and the high-level expression of both CX3CR1 and CX3CL1/Fractalkine has been reported in the central nervous system. [17]. 

In the presence of inflammatory stimuli, the interaction between CX3CL1/Fractalkine and CX3CR1 serves various functions in regulating immune balance by promoting the migration of monocytes along vascular endothelial cells, expediting the influx of circulating leukocytes into inflamed tissues, and supporting the survival of specific leukocyte populations. Given these functions, it is unsurprising that the CX3CL/Fractalkine–CX3CR1 axis is implicated in the development of numerous inflammatory disorders.

In viral infections, e.g., HIV, COVID-19, influenza, dengue fever, and CMV infection, CX3L1/Fractalkine and CX3CR1+ cells were previously described as important players in immunity [18].

Experimental models demonstrated an increase in CX3CR1 and CX3CL1/Fractalkine expression in hepatic damage, which suggests that regenerating epithelial cells can be both the source and the target for CX3CL1/Fractalkine [19]. However, the levels of CXC3CL1/Fractalkine in the blood plasma of HBV-infected individuals have not been previously analyzed. There are limited existing data on the association between CX3CL1/Fractalkine and fibrosis severity in liver tissue. Considering the fact that CX3CL1/Fractalkine participates in the recruitment of the immune cells responsible for protection against HBV, the goal of this study was to measure its concentrations in the blood plasma of individuals infected with HBV and to assess the role of immune responses caused by this chemokine in liver fibrosis. 

## 2. Materials and Methods

### 2.1. Patients

The study was performed in the time span between 2015 and 2019. The study included 47 HBV patients aged 24–70 with a confirmed HBV infection.

All participants were admitted to the Clinical Infectious Disease Hospital named after S.P. Botkin in Saint Petersburg. Diagnosis was established according to the official World Health Organization (WHO) guidelines [20]. Persistent presence of HbsAg at minimal concentrations of 3.5–4.5 log10 IU/mL for >6 months was considered the main criterion. Other diagnostic criteria included the following HBV-specific serological markers detected in the blood plasma via ELISA: anti-HBs IgGs, anti-HBCor IgGs, HBeAg, and anti-HBe IgGs. Another marker for chronic HBV infection was the quantitative detection of viral DNA with PCR testing, with a viral load above 10^3^ IU/mL.

To recognize and evaluate HBV-specific changes, we added two comparison groups. We included 43 patients from the Clinical Infectious Disease Hospital named after S.P. Botkin with hepatitis C virus (HCV) infection; their demography was compatible with the HBV group. According to the WHO [21], the diagnostic criteria include qualitative ELISA detection of blood plasma anti-HCV Ig (both M and G) and the detection of viral RNA.

Another comparison group included 30 patients with autoimmune liver diseases, i.e., autoimmune hepatitis and primary biliary cholangitis (PBC). These patients were treated in the Clinical Hospital #31. For these patients, all the other causes of liver damage, such as liver-specific infections or toxic damage from substance abuse, were ruled out.

The control group included 32 healthy donors without any clinical or laboratory markers of liver disease or any somatic disease.

Additional diagnostic procedures were performed by the medical staff of the hospital and included physical examination, abdominal ultrasound, liver elastography, and detection of liver enzymes in blood (ALT, AST). The METAVIR scoring system [22] was used to assess the extent of liver fibrosis with elastography. Fibrosis levels range from F0 (indicating no fibrosis) to F4 (indicating cirrhosis) and are mainly determined by the presence of fibrosis in septa. A level of F2 or above is classified as significant fibrosis, whereas F3 or higher is regarded as advanced fibrosis.

Exclusion criteria for this study were as follows: HIV infection, pregnancy, current cancer diagnosis or treatment, and chronic diseases in an acute stage.

Peripheral blood was used for this analysis. Blood samples were collected in vacuum tubes with K_2_EDTA, centrifuged at 350× *g* for 10 min, then transferred to cryotubes and frozen at −80 °C until the analysis. The baseline characteristics of the patients with HBV, HCV, autoimmune liver diseases, and the healthy donors are represented in Table 1.

**Figure 1:**
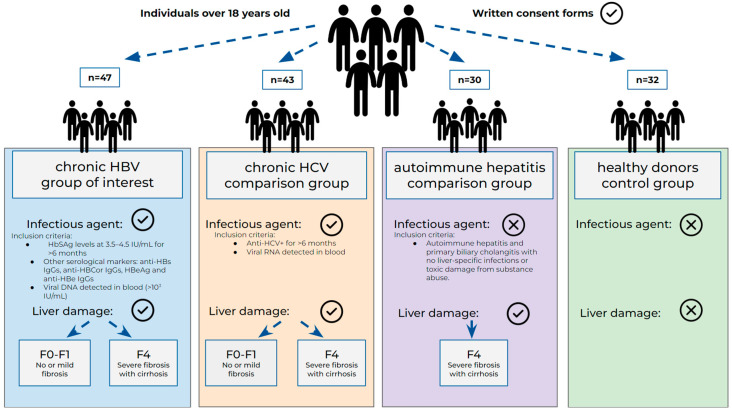
Patients included in the study. HBV—hepatitis B virus, HCV—hepatitis C virus; F0–F1, F4—fibrosis stage based on METAVIR classification.

A visualization of the experimental design is presented in Figure 1.

### 2.2. Cytokine Analysis

We measured the concentrations of CX3CL1/Fractalkine (pg/mL) in the blood plasma with the xMAP technology (Diasorin, Saluggia, Italy) for multiplex analysis using the Milliplex HCYTA-60K-PX48 (Millipore, Burlington, MA, USA) kit. Data collection and analysis were performed with Luminex MAGPIX (Diasorin, Saluggia, Italy). The experimental part of the work was performed at the ‘Cytometry and Biomarkers’ core facility of the Saint Petersburg Pasteur Institute.

### 2.3. Statistical Analysis

Statistical analysis was performed in GraphPad Prism 6.0 (Dotmatics, Boston, MA, USA). As the clinical and laboratory data did not pass the Shapiro–Wilk normality test, we used the following methods of non-parametric statistics: U-test, Mann–Whitney test, and correlational analysis (Spearman’s R). Observations were considered statistically significant at *p*-value < 0.05. Descriptive statistics include medians (Me) and the interquartile range (Q25–Q75). Specificity and sensitivity were measured with receiver operating characteristic (ROC) analysis and the detection of the area under curve (AUC). 

### 2.4. Visualization

For graphs and representation of statistical analysis, we used GraphPad Prism 6.0 (Dotmatics, Boston, MA, USA). For visualization of biological processes, we used the following OpenSource web platforms: Photopea (https://www.photopea.com/, accessed on 24 July 2024) and Bioicons (https://bioicons.com/, accessed on 24 July 2024).

## 3. Results

The results of the CX3CL1/Fractalkine concentration analysis are presented in Figure 1. We compared the groups with different pathologies (HBV, HCV, autoimmune hepatitis) with the healthy donors and included analysis based on fibrosis METAVIR score [22]. We included two polar groups, group F0–F1, which included patients with no or mild fibrosis, whereas F4 suffered from severe fibrosis with septa and/or cirrhosis. 

The comparison showed a statistically significant depletion of CX3CL1/Fractalkine in the samples from HBV-infected individuals with severe liver fibrosis. Although our study included other pathologies with F4 fibrosis, only in HBV did we note a decrease in the CX3CL1/Fractalkine concentrations. CX3CL1/Fractalkine is a key player in liver fibrosis formation. In HCV infection, it is usually a marker of severe cirrhosis and fibrosis [23,24]. The presence of CX3CR1 molecules in the liver tissue ensures protection against fibrosis, whereas the lack of this receptor on the cells leads to a quicker development of liver damage [25]. In non-infectious liver diseases (e.g., PBC), CX3CL1/Fractalkine perpetuates the autoimmune progression of the diseases via TLR-based mechanisms and the recruitment of adaptive immunity cells [26]. 

**Figure 2:**
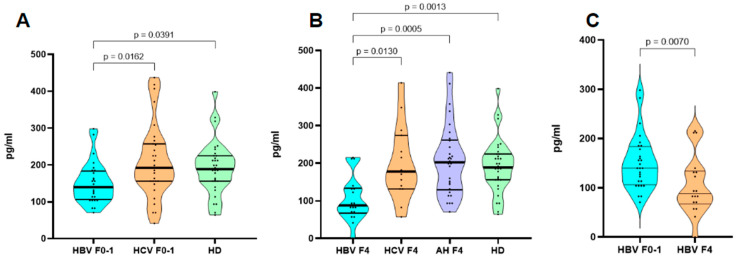
Violin plot representing concentrations of CX3CL1/Fractalkine in blood plasma. HBV—chronic HBV infection (*n* = 47); HCV—chronic HCV infection (*n* = 43); AH—autoimmune hepatitis (*n* = 30), HD—healthy donors (*n* = 32). (**A**) Comparison between groups with F0 (no or mild fibrosis) and healthy donors; (**B**) Comparison between groups with F4 (severe fibrosis/cirrhosis) and healthy donors; (**C**) Comparison between F0–1 and F4 in patients with chronic HBV. Each dot represents one observation; thick black horizontal dashed line represents Median, thin black horizontal sashed line represents interquartile ranges (Q25–Q75). In graphs (**A**,**B**), we applied the ANOVA (Kruskal–Wallis test), while for graph (**C**), we utilized the Mann–Whitney U test.

Despite causing fibrosis in liver tissue, HBV and HCV demonstrated different CX3CL1/Fractalkine profiles. We conducted ROC analysis to evaluate the potential of CX3CL1/Fractalkine in the blood plasma as a differential marker of etiology behind fibrosis (Figure 2). The results indicate that the CX3CL1/Fractalkine levels in blood plasma can indeed serve this function for HBV infection.

As such, the following question remained: Do lowered CX3CL1/Fractalkine concentrations hold prognostic value in terms of fibrosis development in liver tissue? We performed an ROC analysis to show that lowered CX3CL1/Fractalkine concentrations hold prognostic value in terms of fibrosis development in liver tissue. Our findings suggest that the decreased levels of CX3CL1/Fractalkine are associated with an increased risk of fibrosis progression, making it a potential prognostic marker for liver fibrosis development. 

**Figure 3:**
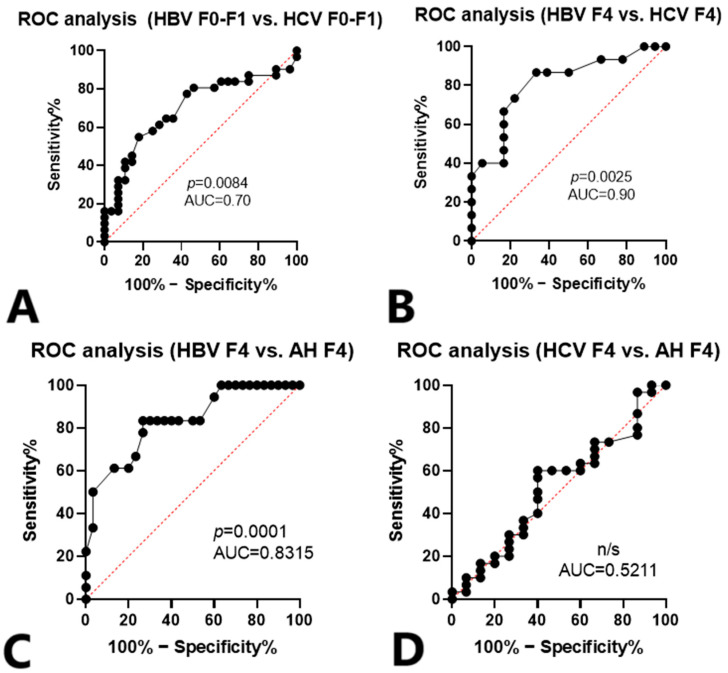
Receiver operator curves with sensitivity and specificity for CX3CL1/Fractalkine concentrations. (**A**)—HBV infection vs. HCV infection in patients with F0–F1 fibrosis. (**B**)—HBV infection vs. HCV infection in patients with F0–F1 fibrosis. (**C**)—HBV infection vs. autoimmune hepatitis (AH), F4 fibrosis. (**D**)—HCV infection vs. AH, F4 fibrosis.

**Figure 4:**
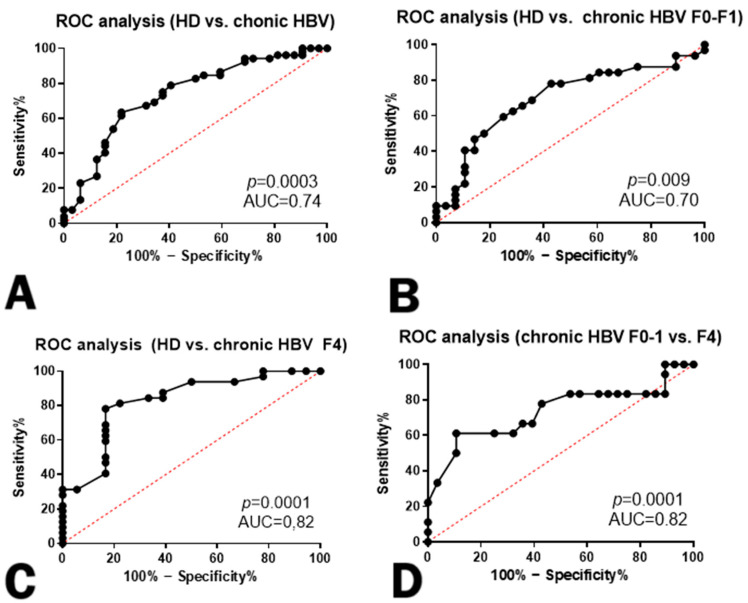
Receiver operator curves with sensitivity and specificity for CX3CL1/Fractalkine concentrations in healthy donors and patients with chronic HBV infection based on fibrosis severity. (**A**)—Healthy donors (HD) and chronic HBV infection; (**B**)—Healthy donors (HD) and patients with no or mild fibrosis F0–1; (**C**)—Healthy donors (HD) and patients with severe fibrosis F4; (**D**)—Patients with mild (F0–1) and severe (F4) HBV-associated fibrosis.

For the next step, we compared HBV-infected individuals based on their infection stage and METAVIR fibrosis classification [20]. Results of ROC analysis are presented in Figure 3 and Figure 4.

We also performed correlational analysis between plasma CX3CL1/Fractalkine levels, ALT, AST, and, where applicable, the viral load. Although prominent as a fibrosis marker, CX3CL1/Fractalkine showed no correlations with these markers.

## 4. Discussion

Our research indicates that the plasma levels of CX3CL1/Fractalkine vary in the HBV-infected group compared to healthy donors and other cohorts. Even at the initial stages of liver fibrosis due to HBV, the CX3CL1/Fractalkine levels are reduced when compared to both HCV patients and healthy individuals. In cases of severe liver fibrosis, CX3CL1/Fractalkine levels are significantly higher than those in healthy donors, as well as in HCV and autoimmune hepatitis patients, where the levels remain within the normal range.

In the context of HBV infection, there is a limited number of studies concerning CX3CL1/Fractalkine. This is especially true for the assessment of CX3CL1/Fractalkine in the blood plasma or serum, despite their availability for assessing systemic immune responses. One such study by Zhu B. et al. investigated multiple cytokines, including CX3CL1/Fractalkine, in association with the outcome of an HBV infection [27]. In this study, it was shown that individuals who did not survive an HBV infection had elevated plasma levels of CX3CL1/Fractalkine in comparison to those who survived. This study, although it established a connection between the CX3CL1/Fractalkine levels and the disease outcome, has certain limitations—the absence of data on liver fibrosis infection and the lack of comparison with healthy donors.

It is worth mentioning that HBV is very heterogenic. Kondo J. et al. demonstrated that different HBV genotypes influence the expression of CX3CL1/Fractalkine [28]. Currently, HBV has nine genetic types that differ in nucleotide sequences for more than 8% and 35 subgenotypes with a 4–7.5% variation [29,30,31]. Moreover, the genetic variants of HBV show a specific distribution based on the region. In Russia, genotype D of HBV is more prevalent than other genotypes, whereas in China and Southeast Asia, genotypes B and C are more dominant [32]. It was shown that the CX3CL1/Fractalkine mRNA expression varied based on the HBV genotype, where the expression levels were higher in genotype C than in genotype B or even in HCV infection. Similar results were seen for the blood serum in patients infected with different genetic variants of HBV. The authors suggested that differences in the HBV genotypes can affect CX3CL1/Fractalkine expression. It is possible that our findings regarding the lowered concentrations of CX3CL1/Fractalkine can be explained by genotype D prevalence in Russia. This finding requires a more detailed approach.

The role of CX3CL1/Fractalkine in severe fibrosis is particularly noteworthy when contrasted with early fibrosis. ROC analysis has validated the significance of this marker in distinguishing the severity of fibrosis. 

Cells with cytotoxic activity are highly important for defense against viral infections, including HBV infection [33]. Based on the balance of activating and inhibitory signals, along with the surrounding cytokine environment, cytotoxic cells exhibit at least the following two key functions: they can generate a range of antiviral and immunoregulatory cytokines. Additionally, they have the capability to directly eliminate target cells by releasing perforin and granzymes.

Decreased concentrations of CX3CL1/Fractalkine, specifically in HBV, can be explained by the evading mechanisms of the HBV virus towards cytotoxic cells. It has been shown that an HBV infection can affect cytotoxic cells, potentially leading to the deterioration of their functional activity, for instance, via the activation of T regulatory cells and the consequent production of IL-10 [34,35]. 

**Figure 5:**
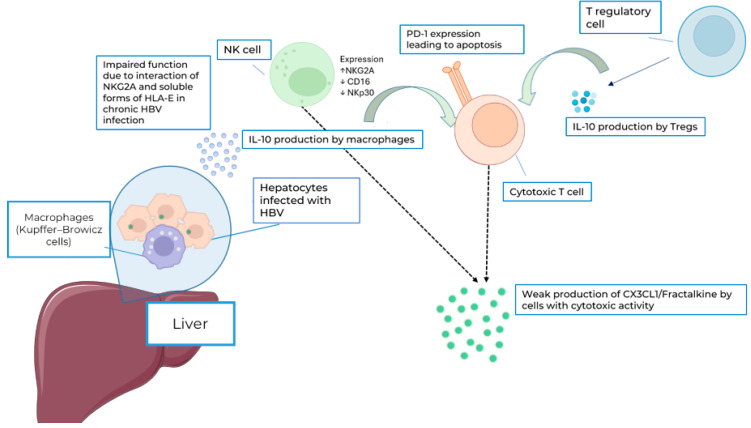
Possible mechanism behind low plasma CX3CL1/Fractalkine concentrations in HBV infection. Due to impaired function of NK cells caused by high viral load and IL-10-induced PD-1 hyperexpression on CTLs, production of CX3CL1/Fractalkine is weakened. Blue arrows represent normal production of IL-10, whereas dotted lines stand for impaired production.

An HBV infection also stimulates the expression of PD-1 molecules on cytotoxic cells [36] and leads to impaired cytolytic function of NK cells and CTLs [37]. The frequency of PD-1 in CX3CR1+ CD8+ T cells in the peripheral blood of chronic HBV-infected individuals was shown to be significantly higher than in chronic HCV-infected individuals [28]. One of the reasons for the NK cell impairment can be the interaction of the highly expressed checkpoint inhibitor molecule NKG2A with a non-classical HLA-E. Expression of the latter is also increased in chronic HBV infection and severe fibrosis [38]. We present our view on plasma CX3CL1/Fractalkine depletion in an HBV infection in Figure 5. This figure encompasses various factors in addition to CX3CL1/Fractalkine, such as IL-10 and PD1/PDL1 expression. These elements could serve as potential targets for future research.

## 5. Conclusions

Overall, the results showed the predictive value of CX3CL1/Fractalkine in terms of fibrosis/cirrhosis development in chronic HBV infection. Although there is still a lot to explore about this chemokine, there is grounds for the discussion of the role of CX3CL1/Fractalkine in HBV-associated processes in the liver. For example, what comes first? Does immune cell impairment in HBV and CX3CL1/Fractalkine depletion lead to fibrosis and damage to liver tissue? Or is it the other way around? Fully tracing the dependencies and timing of these molecular changes, and their associated pathophysiology, requires further study. 

### Study Limitations

Single time point analysis may not be fully representative in terms of CX3CL1/Fractalkine dynamics in blood plasma. We realize that our choice of blood plasma sample design, based on both the ethical considerations and methodology of the study, may limit the generalizability of the findings. Due to the challenges that were present in obtaining biological samples from patients with acute-stage chronic hepatitis, our sample size is relatively small. We kindly ask the readers to consider this when evaluating the results of this study.

## Figures and Tables

**Table 1:** Clinical characteristics of patients included in the study.

	HBV		HCV		Autoimmune Liver Diseases	Healthy Donors
METAVIR liver fibrosis	F0–1	F4	F0–1	F4	F4	n/a
*n*	28	19	30	13	30	32
Male/Female, %	10/18 35%, 65%	9/10 47%, 53%	12/18 40%, 60%	5/8 38%, 62%	2/28 7%/93%	15/17 46%/54%
Age (Mean ± SD)	37.7 ± 8.7	60 ± 9.2	39.0 ± 10.4	41.0 ± 9.1	59.2 ± 9.1	32.0 ± 6.4
ALT, Me (min–max)	23.46 (7.66–72) U/L	21 (8–286) U/L	57.8 (7–198) U/L	54 (50–148) U/L	58 (21–175) U/L	19 (12–33) U/L
AST, Me (min–max)	22 (8–44) U/L	49 (11–327) U/L	44.4 (17–143) U/L	36 (29–49) U/L	68 (31–207) U/L	20 (14–25) U/L
AST/ALT (De Ritis) ratio (Mean ± SD)	0.89 ± 0.33	2.74 ± 3.8	0.87 ± 0.65	1.01 ± 0.65	1.32 ± 0.75	1.33 ± 0.42

## Data Availability

The original contributions presented in the study are included in the article, and further inquiries can be directed to the corresponding author.
